# Validation of a One-Step Reverse Transcription-Droplet Digital PCR (RT-ddPCR) Approach to Detect and Quantify SARS-CoV-2 RNA in Nasopharyngeal Swabs

**DOI:** 10.1155/2021/8890221

**Published:** 2021-03-02

**Authors:** Catia Mio, Adriana Cifù, Stefania Marzinotto, Barbara Marcon, Corrado Pipan, Giuseppe Damante, Francesco Curcio

**Affiliations:** ^1^Department of Medicine (DAME), University of Udine, Udine, Italy; ^2^Department of Laboratory Medicine, University Hospital of Udine, Udine, Italy

## Abstract

**Background:**

Severe acute respiratory syndrome coronavirus 2 (SARS-CoV-2) infection has rapidly spread worldwide from the beginning of 2020. Quantitative reverse transcription-PCR (RT-qPCR) is, to this day, the preferred methodology for viral RNA detection, even if not without problems. To overcome some of the limitations still existing for the detection and quantification of nucleic acids in various applications, the use of one-step reverse transcription-droplet digital PCR (RT-ddPCR) has been established. The purpose of this study was, then, to evaluate the efficacy of ddPCR for the detection of SARS-CoV-2 RNA in nasopharyngeal swabs, optimizing the detection of low-viral load-burdened samples.

**Methods:**

The RT-ddPCR workflow was validated for sensitivity, specificity, linearity, reproducibility, and precision using samples from 90 COVID-19-infected patients referred to the Department of Laboratory Medicine of the University Hospital of Udine (Italy).

**Results:**

The present study shows that RT-ddPCR allows the detection of as low as 10.3 copies of a SARS-COV-2 *E-gene* per sample with a higher level of accuracy and precision, especially at low concentration.

**Conclusion:**

During the postpeak phase of the SARS-CoV-2 pandemic, it is essential to rely on a highly robust molecular biology method to identify infected subjects, whether they have symptoms or not, in order to prepare appropriate containment measures.

## 1. Introduction

The emergence of severe acute respiratory syndrome coronavirus 2- (SARS-CoV-2-) related disease (COVID-19) in China at the end of 2019 caused a major global outbreak and still represents a serious public health issue. Indeed, towards the end of January 2020, the World Health Organization (WHO) declared COVID-19 as the sixth public health emergency of international concern [1]. The so-called SARS-CoV-2 is a single-stranded RNA virus belonging to the genus Betacoronavirus. Multiple sequence alignments revealed that SARS-CoV-2 is closely related to bat-derived SARS-like coronaviruses (88–89% similarity), but when compared to severe acute respiratory syndrome coronavirus (SARS-CoV) and Middle East respiratory syndrome coronavirus (MERS-CoV), SARS-CoV-2 showed less genetic similarity (79% and 50%, respectively) [2]. The associated disease, called COVID-19, is characterized by fever, fatigue, dry cough, pharyngodynia, shortness of breath, headache, chest tightness, chest pain, and myalgia [3]. In some patients, the symptomatology worsens rapidly leading to acute respiratory distress syndrome (ARDS) [4]. A variable proportion of infected subjects, estimated to be around 20–25%, does not show any symptoms, making it difficult to limit the infection, since these patients are capable to spread the virus and may represent a population that can be easily neglected in the prevention of epidemics. Indeed, public health authorities would need to implement rapid and sensitive diagnostic tools for patients' management in the shortest possible time. According to the World Health Organization (WHO), the current gold standard for the diagnosis of SARS-CoV-2 infection is based on quantitative reverse transcription-PCR (RT-qPCR), which can detect SARS-CoV-2 nucleic acid patient samples.

Droplet digital PCR (ddPCR) is a variant of the emulsion-based PCR technology. It is a promising, ultrasensitive approach, capable of compartmentalizing samples into millions of picoliter droplets containing single-nucleic acid (NA) molecules and analyzing the terminal fluorescence of each droplet after parallel amplification [5]. Moreover, it eliminates the need for a standard curve as for quantitative PCR (qPCR) [5, 6]. This cutting-edge technology has quickly entered into clinical practice, providing useful indications for prognostic evaluation, monitoring, and characterization of diseases but also for the detection of nucleic acids of pathogens [7, 8]. Furthermore, several data on the development of the reverse transcription-ddPCR (RT-ddPCR) method have been produced, demonstrating that this technique has a higher precision and improved sensitivity to detect rare targets at low copy numbers than RT-qPCR.

During the postpeak phase of the SARS-CoV-2 pandemic, it is mandatory to rely on a highly robust molecular biology technique to detect infected subjects, whether symptomatic or not, to set up appropriate containment measures. The aim of this study was to validate and optimize the detection of low-viral load-burdened patients coupling the already available primers and probe sets and the RT-ddPCR technique, identifying the most suitable workflow.

## 2. Materials and Methods

### 2.1. Patient Samples

SARS-CoV-2 samples were collected from 90 symptomatic, paucisymptomatic, or asymptomatic patients by nasopharyngeal swab, referred to the Department of Laboratory Medicine, University Hospital of Udine, Italy, and reported to be positive by standard molecular biology procedures (RT-qPCR). For swab collection, transportation, and long-term storage, UTM® tubes (COPAN Diagnostics) were used, according to manufacturer's instructions. Ethical approval was obtained from the Medical Research Ethics Committee of the Region Friuli Venezia Giulia, Italy (Consent CEUR-2020-Os-033).

### 2.2. RNA Extraction from Nasopharyngeal Swab

For the automatic extraction with the ELITe InGenius® SP200 (ELITechGroup) system of SARS-CoV-2 RNA, 200 *μ*L of the medium from UTM® tubes containing 3 mL was used, following the manufacturer's instructions. Samples were eluted in 100 *μ*L elution buffer and used as the template for downstream analysis. Extracted RNA was stored at −80°C.

### 2.3. One-Step Reverse Transcription-Droplet Digital Polymerase Chain Reaction (RT-ddPCR)

5′6-FAM/3′BHQ-1®-conjugated assays were used for viral load assessment by the One-Step RT-ddPCR Advanced Kit for Probes (Bio-Rad). Oligonucleotides are listed in Table 1 and were purchased from Merck KGaA. Briefly, 5 *μ*L of ddPCR™ Supermix for Probes (No dUTP), 900 nM primers and 250 nM probes, 15 mM DTT, 20 U/*μ*L reverse transcriptase, 2 *μ*L sample, and nuclease-free water were mixed in a total volume of 20 *μ*L. Samples were mixed with Droplet Generator Oil for Probes (Bio-Rad) and droplets were generated with the automated droplet generator QX200™ droplet generator (Bio-Rad). PCR amplification was performed on the Veriti® Thermal Cycler (Thermo Fisher Scientific). Amplification was performed at 50°C for 60 min for reverse transcription, 95°C for 10 min for enzyme activation, and followed by 45 cycles of 95°C for 30 s, 55°C for 60 s, and then 98°C for 10 min for enzyme deactivation. Droplets were read on the QX200™ droplet reader (Bio-Rad) and reactions with less than 10,000 droplets were repeated. Primers and the 5′HEX/3′ BHQ-1®-conjugated probe for *RNaseP* human gene were evaluated to verify the adequacy of RNA isolation. Data were analyzed using the QuantaSoft™ 1.7.4 Software (Bio-Rad). For all experiments in this work, the same thresholds were used for FAM and HEX signals. These were determined by running in a multiplex positive control (lyophilized positive control from the LightMix® Modular SARS and Wuhan CoV E-gene from Roche) and no template control samples.

### 2.4. Quantitative Reverse Transcription-Polymerase Chain Reaction (RT-qPCR)

To detect viral RNA, LightMix® Modular SARS and Wuhan CoV E-gene (Roche) was used as previously described [9]. RT-qPCR was performed by the LightCycler® 480 II Instrument (Roche) and absolute quantification was assessed by the LightCycler® 480 II System (Roche). The estimated viral copy number extracted from the Cq values by RT-qPCR was calculated based on the linear regression of a serial dilution made with the synthetic control for the *E gene* supplied by Roche in the LightMix® Modular SARS and Wuhan CoV E-gene kit.

### 2.5. Limit of Blank (LoB) and Limit of Detection (LoD)

The Limit of Blank (LoB) and Limit of Detection (LoD) have been evaluated to avoid false-positive and false-negative results and to reliably and critically describe the minimum viral load that can be detected with this method. The Limit of Blank (LoB) has been established as the highest number of viral copies that can be measured when no template control (NTC) replicates are tested. The LoB was established using the signal-to-noise ratio, i.e., by measuring false-positive events from 40 wells of NTC for each primer and probe set (see Table 1), using the following formula: LoB = Mean_NTC_ + 1.645∗SD_NTC_ as previously described [10, 11]. The LoD represents the lowest amount of analyte that can be reliably detected, i.e., the lowest number of copies that can be clearly distinguished from the background with 99% probability, ensuring a false-positive rate ≤ 5%. In our study, the LoD was calculated as: LoD = Mean_NTC_ + 3∗SD_low_, being SD_low_ the standard deviation of low-concentration samples [11–13]. The LoB and LoD have been established for all the oligonucleotides listed in Table 1.

### 2.6. Limit of Quantification (LoQ)

The LoQ represents the lowest amount of analyte that can be reliably quantified with precision and accuracy. It was computed through the signal-to-noise method as LoQ = Mean_NTC_ + 10∗SD_low_. In addition, for the *E gene* alone, the LoQ was further validated by 7 serial dilutions (fivefold each) of a SARS-CoV-2-positive sample, with each dilution being repeated ten times (see Section 2.10). The concentration that had a coefficient of variation (CV) of less than 30% was used as the LoQ [10, 14, 15].

### 2.7. Agreement

To evaluate the agreement between our RT-ddPCR and the gold standard (RT-qPCR), Bland-Altman analysis was used. A total of 60 samples were used for this purpose, divided in 30 positive and 30 negative samples for SARS-CoV-2. To evaluate the distribution of the data, the Shapiro-Wilk normality test was performed. A comparison of the methods was made using the Spearman rank correlation coefficient analysis. Raw data from the analysis of positive samples with RT-qPCR and RT-ddPCR are presented in Supplementary Table 1.

### 2.8. Accuracy

The accuracy of an analytical procedure is the closeness of the test results obtained by that method to a specific value, be it the true conventional value or the accepted reference one. The evaluation of RT-ddPCR accuracy was performed by testing four different dilutions of a positive sample (Cq 20.7 by RT-qPCR), with 10 replicates each. The positive control was diluted using the buffer in which the viral RNA was extracted in (ELITe InGenius® SP200 system).

### 2.9. Precision

The precision of an analytical procedure is defined as the closeness of measurements of a series obtained from multiple determinations of the same sample under specific conditions. It can be established as repeatability and intermediate precision. Repeatability was assessed by evaluating the variance by analyzing a positive sample at three different dilutions, three times on the same day, using the same equipment and the same batch for each reagent. Intermediate precision was assessed by measuring a positive sample at three different dilutions, three times each day for three consecutive days. The distribution of each series of results was checked with the Shapiro-Wilk test. To assess the equality of variances, the precision was examined using the one-way ANOVA, according to ISO 5725 guidelines [16].

### 2.10. Linearity

The linearity of an analytical procedure is its ability to obtain test results that are directly proportional to the concentration of an analyte in samples within a given range or through well-defined mathematical transformations. The linear range of RT-ddPCR was established with a series of 7-step dilutions. Each dilution was tested in 10 replicates. The relationship between the observed values and the gold standard was examined by linear regression.

### 2.11. Statistics

The statistical analysis was performed with GraphPad Prism 6.0. Grubb's test was used to identify the outliers within replicates. Quantitative variables were analyzed with one-way ANOVA. A *p* value ≤ 0.05 was considered statistically significant. ^∗^*p* < 0.05, ^∗∗^*p* < 0.01, ^∗∗∗^*p* < 0.001, and ^∗∗∗∗^*p* < 0.0001.

## 3. Results

We optimized a RT-ddPCR workflow using the primer and probe sets published in early 2020 by both the German Consiliary Laboratory for Coronaviruses (Charité, Berlin) [17] and the United States Center for Disease Control and Prevention (CDC, Atlanta) [18], which have been approved for COVID-19 *in vitro* diagnostics. Table 1 contains information about the six different primer pairs (named assays) used in this study, which are directed to specific regions of the SARS-CoV-2 genome. The Limit of Blank (LoB) was determined for all assays by analyzing 40 no template control (NTC) replicates. All analytical points showed more than 10,000 droplets and were therefore retained for further analysis. The use of N1 assay was immediately abandoned due to the high rate of primer dimers that create positive amplification in all NTC replicates (data not shown). The Limit of Detection (LoD) was then calculated for all 5 assays, as the mean of NTCs plus three standard deviation of low concentrated samples. As shown in Table 2, the assay targeting the *envelope gene* (*E gene*) presented the lowest LoD (7 copies/reaction) compared to the other assays. To make sure that the patient samples were evaluated in the best possible way, avoiding to lose the low viral loads, we calculated the Limit of Quantification (LoQ), and to do so, we used the assay that evaluates the *E gene* since it is the one that demonstrated the best performance (Table 2).

We then evaluated the sensitivity and specificity of all five assays, testing 30 patient samples, (19 positives and 11 negatives) with standard molecular biology techniques for the detection of SARS-CoV-2 (i.e., RT-qPCR) [19]. With the exception of the N2 assay, all primers and probes showed 100% specificity but a variable degree of sensitivity.  Table 3 summarizes the sensitivity and specificity percentages for all the assays evaluated.

Considering all data collected so far, we continued our workflow validation using primers and probes for the *E gene*, the assay that has shown the lowest LoQ, and the best combination of specificity and sensitivity.

**Figure 1:**
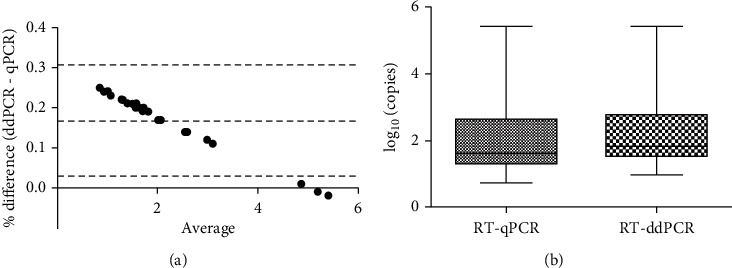
Agreement between RT-qPCR and RT-ddPCR in SARS-CoV-2 RNA detection. (a) Bland-Altman plots representing the agreement between RT-qPCR and RT-ddPCR. Dotted lines represent the upper limit of 95% agreement, the bias, and the lower limit of 95% agreement. (b) Boxplot showing log_10_ of copies of SARS-CoV-2 RNA evaluated with both RT-qPCR and RT-ddPCR. *p* > 0.05 (ns) by Mann–Whitney *U* test.

The Bland-Altman analysis showed that all negative samples presented viral copies below the estimated LoQ. The bias between the measurements was 0.168 (95% CI: 0.030-0.307) with an agreement of 95% (Figure 1). The Shapiro-Wilk normality test showed that the population was not normally distributed (*p* < 0.0001). Spearman's rho test showed a high correlation between the two methods with a coefficient of *r* = 0.9998 (*p* < 0.0001).

The coefficient of determination (*R*^2^) showed that the relation between the expected viral copies (RT-qPCR) and the measured ones (RT-ddPCR) was linear (*R*^2^ = 1.00) and the slope of the fitted line was 0.91. This regression coefficient indicates that RT-ddPCR detects 9% less viral copies than expected. A more accurate estimate of this bias has been obtained by evaluating the accuracy of the method.

The accuracy of the method was assessed analyzing 4 different concentrations with 10 replicates each. The accuracy was expressed as the coefficient of variation (CV). For molecular biology assays, CV values are accepted when they are less than 30% [20]. The results showed that our workflow has a high level of accuracy, with CV < 30% for all the tested dilutions. The data are summarized in Table 4.

Finally, the precision evaluation data are presented in Table 5. The repeatability and intermediate precision have been grouped for each test and analyzed with a unidirectional analysis of the variance (ANOVA). The coefficient of variation for both parameters was <25% for all dilutions analyzed.

## 4. Discussion

The SARS-CoV-2 pandemic posed a major challenge for national and local public health laboratories, which had to be reorganized very quickly to identify infected individuals as soon as possible to enable the development of containment strategies to prevent the spread of the virus. According to the World Health Organization (WHO) and Chinese Center for Disease Control and Prevention (CDC), the current gold standard for the diagnosis of SARS-CoV-2 infection is based on the quantitative reverse transcription-PCR PCR (RT-qPCR) [19], because it has great scalability, it is fast, and it is fairly inexpensive. On the other hand, qPCR results in low precision and accuracy assessing samples with a low viral load, with a sensitivity reported to vary from 30% to 60% depending on the assay [21–24].

Since January 2020, several assays have been proposed for the accurate detection of viral RNA in samples of patient with COVID-19. In this study, we compared the first six published assays to assess their detection performance (in terms of sensitivity and specificity) using samples collected during the first wave of the outbreak in Italy. The results showed that there are substantial differences in the ability to correctly identify weakly positive samples. Moreover, most of these assays showed a high LoQ, which makes them unsuitable for diagnostic purposes. For all these reasons, we validated our RT-ddPCR workflow using primers and probes for the *envelope gene* (*E-gene*), since it was able to discriminate 10.3 copies of the *E-gene* of SARS-CoV-2 from the background, with high sensitivity and specificity (90% and 100%, respectively). Experiments evaluating the SARS-CoV-2 detection rate in 60 patient samples showed a high correlation between RT-ddPCR and RT-qPCR. However, since a high correlation does not necessarily imply that there is a good agreement between the two methods, we performed a Bland-Altman test. The bias between the two methods was 0.1683 and almost all measured points were within the 95% agreement. Taken together, our data suggest that both RT-ddPCR and RT-qPCR can be used for the detection of SARS-CoV-2 RNA. Despite that the two methods showed good agreement and correlation, RT-ddPCR demonstrated a higher level of accuracy and precision, especially at low concentration, i.e., approaching the LoQ. Moreover, it provides an absolute measurement of viral copies without the need to set the threshold level for RT-qPCR. Since precision is a key feature in diagnostic procedures, to further characterize the advantages of our workflow, we calculated repeatability and intermediate precision by testing three different dilutions of a positive sample every day for three consecutive days. The coefficient of variation (CV) was less than 30% in both inter- and intraday measurements indicating that there was no significant difference in results between evaluations.

Unfortunately, all that glisters is not gold. Sample preparation, droplet generation, and detection take a long time and increase the investigation time. In addition, samples with a high viral titer undergo an underestimation of the actual concentration of the nucleic acid, as the droplets are saturated, and the costs of ddPCR are currently higher in comparison with RT-qPCR.

Indeed, in the last few months, several works have underlined the benefits of using the RT-ddPCR for the analysis of samples with a low SARS-CoV-2 load or derived from patient's saliva, comparing both different platforms (i.e., QX200 system from Bio-Rad, TD-1 system from TargetingOne, or Naica system from Stilla Technologies) and using different available kits [21–23, 25, 26]. Despite the diverse approaches, all of them are in agreement with our data in considering RT-ddPCR a highly robust technique for SARS-CoV-2 detection in clinical applications, to be preferred during patients' follow-up, as the monitoring of the correct viral load is essential to contain the infections.

In summary, despite the limitations, RT-ddPCR is a suitable, precise, sensitive, and accurate method for the detection of SARS-CoV-2 RNA, which should be preferred not in mass screening (i.e., exponential phase of the pandemic) but when the correct detection of infected subjects would prevent the possibility of a further wave of infection. We have validated a workflow based on the detection of the *E gene*, which allows to detect as low as 10.3 viral copies. The workflow described in this work is in fact aimed at reducing false negatives, with the possibility of evaluating subjects with a low viral load that would remain undetectable to mass screening with qPCR or accurately tracking the patient viral load before discharging.

## Figures and Tables

**Table 1:** Primers and probes for SARS-CoV-2 detection used in this study.

Target region	Oligonucleotide	Sequence^a^	Reference
E gene	E gene_F	ACAGGTACGTTAATAGTTAATAGCGT	[17]
	E gene_R	ATATTGCAGCAGTACGCACACA	
	E gene_P	6-FAM-ACACTAGCCATCCTTACTGCGCTTCG-BHQ1	
RdRP gene	RdRP gene_F	GTGARATGGTCATGTGTGGCGG	[17]
	RdRP gene_R	CARATGTTAAASACACTATTAGCATA	
	RdRP gene_P2	6-FAM-CAGGTGGAACCTCATCAGGAGATGC-BHQ1	
N gene	N gene_F	CACATTGGCACCCGCAATC	[17]
	N gene_R	GAGGAACGAGAAGAGGCTTG	
	N gene_P	6-FAM-ACTTCCTCAAGGAACAACATTGCCA-BHQ1	
	N2 gene_F	TTACAAACATTGGCCGCAAA	[18]
	N2 gene_R	GCGCGACATTCCGAAGAA	
	N2 gene_P	6-FAM-ACAATTTGCCCCCAGCGCTTCAG-BHQ1	
	N3 gene_F	GGGAGCCTTGAATACACCAAAA	[18]
	N3 gene_R	TGTAGCACGATTGCAGCATTG	
	N3 gene_P	6-FAM-AYCACATTGGCACCCGCAATCCTG-BHQ1	

^a^Y is C/T, R is A/G, and S is C/G.

**Table 2:** Estimated Limit of Blank (LoB), Limit of Detection (LoD), and Limit of Quantification (LoQ) according to the guidelines of the International Conference on Harmonisation (ICH) [27].

Target	Mean_NTC_	SD_NTC_	LOB	LOD	LOQ
E gene	0.54	0.98	2.1	7	10.3
N gene	0.75	2.02	4.1	13	21
RdRP gene	2.16	2.12	5.7	19.1	23.4
N2 gene	1.09	1.34	3.3	11	14.5
N3 gene	2.90	2.58	7.1	24.3	28.7

**Table 3:** Specificity and sensitivity for SARS-CoV-2 assays tested on 30 patient samples.

Assay	Specificity (%)	Sensitivity (%)
*E*	100	95
*N*	100	63
*RdRP*	100	74
*N2*	91	95
*N3*	100	89

**Table 4:** Accuracy of SARS-CoV-2 detection by RT-ddPCR using the *E gene* assay.

Analyte (%)	Mean (copies)	SD (copies)	CV%
0.1	238.6	24.3	10.2
0.04	87.9	14.9	16.9
0.02	42.4	7.4	17.3
0.01	26.8	6.5	24.4

**Table 5:** Inter- and intra-assay precision expressed as coefficient of variation (CV%).

Analyte (%)	Inter-assay precision		Intra-assay precision	
	CV%	*p* value	CV%	SD (copies)
100	5.6	0.980	1.5	0.1180
1	3.3	0.7404	0.6	0.0653
0.1	19.6	0.498	12.5	0.0646

## Data Availability

The data used to support the findings of this study are included within the article.

## Supplementary Materials

Supplementary Materials Supplementary Table 1: raw data of the 30 positive samples used to assess the agreement between RT-qPCR and RT-ddPCR.
