# Supplementary material for: Validation of a One-Step Reverse Transcription-Droplet Digital PCR (RT-ddPCR) Approach to Detect and Quantify SARS-CoV-2 RNA in Nasopharyngeal Swabs
Source: Dis Markers. 2021 Mar 2;2021:8890221. doi: 10.1155/2021/8890221 (PMC7934789; doi:10.1155/2021/8890221)
Supplement: Supplementary Materials — Supplementary Table 1: raw data of the 30 positive samples used to assess the agreement between RT-qPCR and RT-ddPCR. [file 8890221.f1.docx]

**Supplementary Data**

Supplementary Table 1: Raw data of the 30 positive samples used to assessed agreement between RT-qPCR and RT-ddPCR.

| **N** | **RT-qPCR (Cq)** | **RT-ddPCR (copies)** |
| --- | --- | --- |
| #1 | 20.1 | 251189 |
| #2 | 20.84 | 154373 |
| #3 | 21.97 | 73403 |
| #4 | 34.13 | 25 |
| #5 | 34.03 | 26 |
| #6 | 35.21 | 12 |
| #7 | 32.7 | 63 |
| #8 | 32.64 | 66 |
| #9 | 33.3 | 43 |
| #10 | 35.06 | 13 |
| #11 | 32.32 | 81 |
| #12 | 32.88 | 56 |
| #13 | 32.42 | 76 |
| #14 | 33.18 | 46 |
| #15 | 29.7 | 454 |
| #16 | 27.98 | 1408 |
| #17 | 28.29 | 1148 |
| #18 | 29.83 | 417 |
| #19 | 34.87 | 15 |
| #20 | 31.46 | 143 |
| #21 | 35.33 | 11 |
| #22 | 27.91 | 1474 |
| #23 | 33.74 | 32 |
| #24 | 28.33 | 1118 |
| #25 | 33.1 | 48 |
| #26 | 33.55 | 36 |
| #27 | 32.45 | 74 |
| #28 | 33.09 | 49 |
| #29 | 32.67 | 64 |
| #30 | 31.6 | 130 |
